# StemBell Therapy Does Not Significantly Affect Atherosclerotic Plaque Characteristics in a Streptozotocin-Induced Diabetes Mellitus Mouse Model

**DOI:** 10.3390/biology14091130

**Published:** 2025-08-26

**Authors:** Amber Korn, Suat Simsek, Mitchell D. Fiet, Ingeborg S. E. Waas, Klazina Kooiman, Hans W. M. Niessen, Paul A. J. Krijnen

**Affiliations:** 1 Department of Pathology, Amsterdam University Medical Centres (AUMC), Location VUmc, 1081 HV Amsterdam, The Netherlands; 2 Department of Pathology, Amsterdam University Medical Centres (AUMC), Location AMC, 1105 AZ Amsterdam, The Netherlands; 3 Amsterdam Cardiovascular Sciences, 1081 HV Amsterdam, The Netherlands; 4 Department of Internal Medicine, Northwest Clinics, 1817 MS Alkmaar, The Netherlands; 5 Department of Internal Medicine, Amsterdam University Medical Centres (AUMC), Location VUmc, 1081 HV Amsterdam, The Netherlands; 6 Biomedical Engineering, Department of Cardiology, Cardiovascular Institute, Erasmus MC, 3015 GD Rotterdam, The Netherlands; 7 Department of Cardiac Surgery, Amsterdam University Medical Centres (AUMC), Location VUmc, 1081 HV Amsterdam, The Netherlands

**Keywords:** adipose tissue-derived stem cells, atherosclerosis, diabetes mellitus, mesenchymal stem cell therapy, ultrasound-activated microbubbles

## Abstract

Aims: Diabetes mellitus (DM) increases cardiovascular risk by inducing atherosclerotic plaque instability. StemBell therapy (i.e., adipose tissue-derived stem cells (ASCs) coupled to ultrasound-activated microbubbles) previously improved plaque stability in non-DM ApoE^−/−^ mice. Here, we investigated the effect of StemBell therapy on atherosclerotic plaque characteristics in a streptozotocin-induced DM mouse model. Methods: DM was induced in male C57BL/6 ApoE^−/−^ mice (*n* = 18) via intraperitoneal streptozotocin (STZ) injection (0.05 mg/g bodyweight) for 5 consecutive days. Eight weeks after the first STZ injection, the mice received either 5 × 10^5^ StemBells or vehicle intravenously. Due to unexpected mortality, the experiment was halted and restarted in week 9 with a final reduced dose of 1.25 × 10^5^ StemBells to avoid complications. The effect of StemBell therapy on plaque characteristics was determined 4 weeks post-treatment in five vehicle-treated and five StemBell-treated mice via (immuno)histochemical analyses. Furthermore, plasma monocyte subsets within 3 days pre- and 3 days post-treatment, and 3 weeks post-treatment, were studied via flow cytometry. Results: StemBell therapy did not significantly affect atherosclerotic plaque size or intra-plaque inflammation. StemBell-treated mice had less intra-plaque Ly6G+ neutrophils (0.4 ± 0.5%) and intra-plaque Mac3+ pan-macrophages (17.7 ± 3.4%), but more CD163+ anti-inflammatory M2 macrophages (*p* = 0.5) compared to vehicle-treated mice, although this was non-significant. Conclusions: StemBell therapy did not significantly affect atherosclerotic plaque size or intra-plaque inflammation in a streptozotocin-induced DM mouse model. Future research is essential to explore the potential and limitations of StemBell therapy in DM-related atherosclerosis. The higher mortality of StemBell therapy in diabetic mice compared to the previous non-diabetic mice also warrants further investigation.

## Simple Summary

Cardiovascular disease remains the main cause within the diabetic population, of which atherosclerosis is a major contributor. Atherosclerotic plaque development is driven through cholesterol accumulation and chronic inflammation, and exacerbated by diabetes mellitus (DM) through hyperglycaemia and systemic inflammation. Stabilising plaques may aid in reducing the risk of cardiovascular complications in the diabetic population. A promising therapy is the application of mesenchymal stem cells (MSC), particularly those derived from adipose tissue (ASC), as they have demonstrated therapeutic potential due to their immunomodulatory capabilities. We previously enhanced the effect of these ASCs by coupling them to ultrasound-activated microbubbles, so that the ASCs can be guided towards a specific location. This StemBell technology, as we termed it, improved plaque stability and intra-plaque inflammation in non-DM atherosclerotic mice. Here, we applied StemBells in DM atherosclerotic mice to assess if similar improvements were obtained. However, due to unexpected mortality the Stembell dose had to be lowered 4-fold compared to the previous study in non-DM mice. We did not find significant changes in plaque characteristics, but due to study limitations we cannot draw definitive conclusions from our findings in regard to applying StemBell technology in DM models, and further research is required to develop StemBell technology for clinical use.

## 1. Introduction

Atherosclerosis is a major contributor to cardiovascular disease, which remains the leading cause of morbidity and mortality among individuals with diabetes mellitus (DM) [1,2,3]. The development of atherosclerotic plaques results from chronic cholesterol accumulation within the vessel wall, followed by immune cell infiltration that initiates chronic inflammation. Plaques that are unstable—often identified by a thin fibrous cap and/or heightened inflammatory activity—are more prone to rupture, potentially leading to thrombotic events such as myocardial infarction or stroke [4]. Plaque instability is often aggravated by DM risk factors, including persistent hyperglycaemia and systemic inflammation, as previously observed in both stable DM cases [5] and those with acute coronary syndrome [6]. Additionally, these risk factors result in a pro-inflammatory imbalance of circulating monocyte subsets, further exacerbating atherosclerotic plaque development [7]. Plaque-stabilising strategies may reduce rupture risk and, consequently, the incidence of thrombotic complications.

Mesenchymal stem cells (MSCs) have demonstrated therapeutic potential in a range of cardiovascular conditions, including atherosclerosis [8,9,10]. They can be sourced from tissues such as bone marrow and adipose tissue, and are capable of modulating immune responses, for instance by stimulating macrophage transition to the anti-inflammatory M2 subtype and by secreting anti-inflammatory cytokines [10,11,12]. MSCs were traditionally sourced from the bone marrow (BMSCs), but this harvesting procedure remains invasive and typically has a relatively low yield. Therefore, the use of adipose tissue-derived MSCs (ASCs) is gaining attention due to their abundance and ease of extraction without compromising their immunomodulatory capacities [11,12], making them more suitable for clinical applications. Preclinical studies have shown that BMSCs can reduce both plaque size and instability in non-DM models [8,13]. Moreover, systemic BMSC administration improved hyperglycaemia and nephropathy in diabetic mice [14]. Despite growing interest in DM-related complications (i.e., nephropathy, neuropathy, and retinopathy) and the apparent potential of MSC in DM treatment [15], the specific impact of MSC-based therapies on aortic plaque development in a diabetic context remains unexplored.

**Figure 1:**
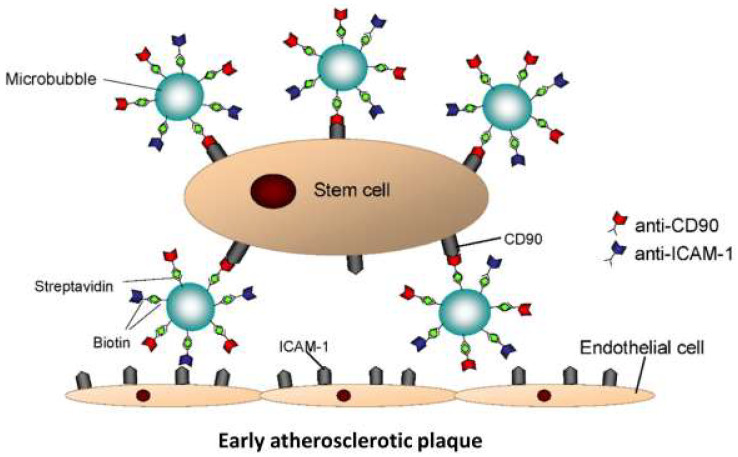
Schematic drawing of a StemBell: a stem cell/microbubble complex that is coupled via streptavidin–biotin–antibody bridging. Image adjusted from Woudstra et al. [16] with permission.

Recently, we introduced the so-called StemBell technology to facilitate targeted delivery of ASCs to sites of vascular injury in vivo [16] (Figure 1). In this approach, ASCs were coupled to ultrasound-activated microbubbles via antibodies against ASC marker CD90. These microbubbles are further functionalized with antibodies against intracellular adhesion molecule-1 (ICAM-1), which is expressed on activated endothelial cells in atherosclerotic plaques [17]. The resulting ASC/microbubble complexes—referred to as StemBells—can be directed to specific vascular sites through the application of localised ultrasound, which propels them toward vessel walls via acoustic radiation force [16]. StemBell therapy previously stabilised aortic plaque morphology in non-diabetic ApoE^−/−^ mice, characterised by a thickened fibrous cap and reduced intra-plaque inflammation, including a decrease in pro-inflammatory macrophage content and an increase in inflammation-resolving macrophages [18]. These findings were accompanied by a positive trend in anti-inflammatory monocytes in the blood. Hence, StemBells may offer a promising therapeutic approach to combat macrovascular complications in diabetes.

In this study, we aimed to validate targeted StemBell therapy in a streptozotocin-induced diabetic ApoE^−/−^ mouse model as a novel therapeutic tool to treat macrovascular inflammation in diabetes. We hypothesised that the immunoregulatory properties of StemBell therapy would lead to improvements in atherosclerotic plaque morphology. Using (immuno)histochemistry, endothelial ICAM-1 expression and the presence of intra-plaque inflammatory cells (i.e., macrophages, lymphocytes, neutrophils, and mast cells) were determined in aortic atherosclerotic plaques. Additionally, we assessed the influence of StemBell therapy on peripheral monocyte populations using flow cytometry.

## 2. Methods

### 2.1. Stem Cell Isolation

As previously described, the stromal vascular fraction (SVF) was harvested from adipose tissue that was isolated from the abdominal subcutis and inguinal fat depots of 5–7 weeks old healthy male C57/Bl6 mice (*n* = 9; Charles River, ‘s Hertogenbosch, The Netherlands) and preserved in liquid nitrogen [19]. One week before administration, the SVF was thawed and seeded in selective MesenCult Basal Medium complemented with 10% MesenCult Supplement and 0.1% MesenPure (Stemcell Technologies, 05513, Vancouver, BC, Canada) to promote the expansion of the adipose tissue-derived stem cells (ASCs). The ASCs were cultured at 37 °C with medium changes every 2–3 days. On the day of treatment, confluent ASCs were harvested with trypsin/EDTA, resuspended in serum-free Dulbecco’s Modified Eagle Medium (DMEM), and adjusted to a final concentration of 5 × 10^5^ ASCs/100 μL.

### 2.2. StemBell Preparation

StemBells were prepared as described previously [18]. In short, 500 μL biotinylated microbubbles (1 × 10^9^ microbubbles/mL) were washed with phosphate-buffered saline (PBS), using a sterile 5 mL syringe with a tap. Each washing step involved adding circa 1 mL PBS to the microbubbles in the syringe, centrifuging the syringe at 400× *g* at 4 °C for 1 min, and removing most of the fluid with the tap. Next, 30 μL 1 mg/mL streptavidin (Sigma Aldrich, S4762, Amsterdam, The Netherlands) was added and incubated for 30 min on ice, followed by another PBS wash. Microbubbles were loaded with antibodies against stem cell marker CD90 and activated endothelial cell marker ICAM-1 [18], via incubation with 10 μL of biotinylated monoclonal anti-CD90 (Abcam, ab25285, Amsterdam, The Netherlands) and 10 μL of biotinylated monoclonal anti-ICAM-1 (Abcam, ab25007, Amsterdam, The Netherlands) for 40 min on ice. The dual-targeted microbubbles were washed with PBS and resuspended in 200 μL PBS. Next, 800 μL ASC suspension was added to obtain the desired concentration of 5 × 10^5^/100 μL (1:100 ASC:microbubble ratio) StemBells in serum-free DMEM. After a 30 min incubation at room temperature under continuous rotation, the StemBell assemblage was completed.

### 2.3. Animal Procedures

**Figure 2:**
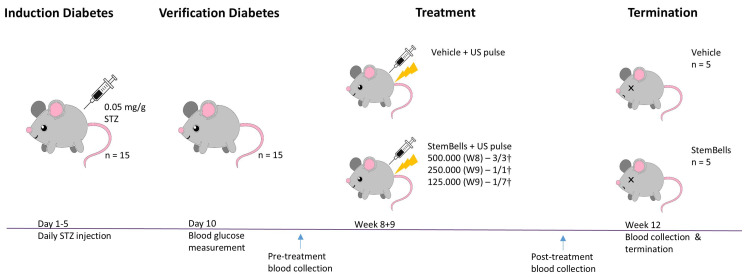
Schematic overview of animal experiment. Diabetes was induced in male C57/Bl6 ApoE^−/−^ mice via administration of 0.05 mg/g bodyweight STZ on 5 consecutive days and confirmed with blood glucose measurements on day 10. After 8 weeks, vehicle solution with or without 1.25 × 10^5^ StemBells was administered intravenously under 2% isoflurane anaesthesia, followed by an ultrasound pulse of 1 min. Injection with 500.000 and 250.000 StemBells resulted in 100% mortality (indicated by †), so the dose was altered to 125.000 StemBells with a 14% mortality. This resulted in a vehicle-treated group (*n* = 5) and a Stembell-treated group (*n* = 5). Within 3 days pre- and post-treatment, and immediately prior to termination 3 weeks after treatment, blood samples were collected from the tail vein or via cardiac punction, respectively.

Diabetes mellitus (DM) was induced in male C57BL/6 ApoE^−/−^ mice aged 7–9 weeks (*n* = 18; Charles River, the Netherlands) in week 0 by intraperitoneal administration of 0.05 mg/g bodyweight streptozotocin (STZ) for 5 consecutive days, as described previously [18,20] (Figure 2). Blood glucose levels were assessed 10 days following initial STZ injection (i.e., in week 1) to verify DM induction, which was defined as a blood glucose concentration exceeding 10 mmol/L (Table 1). All mice developed DM (*n* = 18). The mice received a standard chow diet ad libitum for the entire study duration. This ApoE^−/−^ mouse model is a robust model that induces accelerated development of early atherosclerotic plaques 3 weeks post-STZ administration [21,22]. Body weight was recorded 2–3 times/week from the initiation of STZ administration (start) until the study endpoint (end). StemBell therapy or vehicle solution (i.e., serum-free DMEM) was applied 8 weeks after initial STZ injection, following a previously established protocol [18]. In short, 2% isoflurane was applied to anaesthetise the mice, after which vehicle solution with or without 5 × 10^5^ StemBells was administered via slow intravenous injection into the lateral tail vein using a 25G needle and 1 mL syringe (Terumo, MDSS01SE, Leuven, Belgium). Transthoracic ultrasound was applied immediately thereafter: the transducer (Panametrics, V303-SU, Billerica, MA, USA) was positioned parasternal and directed at the anterior wall of the heart, coupled to a waveform generator (Agilent, 33220A, Middelburg, The Netherlands) and a linear 60 dB power amplifier (Amplifier Research, 150A100B, Paris, France). In this manner, 1MHz ultrasound pulse with a peak negative acoustic pressure of 100 kPa and a 1 kHz pulse repetition frequency (10% duty cycle) was applied for 1 min [23]. We initially injected 5 × 10^5^ StemBells per mouse as per the previous protocol [18]. However, breathing distress and respiratory collapse resulted in death immediately after injection with StemBells (but not vehicle solution). Due to these unforeseen detrimental effects, the experiment was resumed a week later in consultation with the animal ethics committee, applying a reduced dose of 1.25 × 10^5^ StemBells per mouse. Finally, one mouse of the StemBell-group was excluded due to lack of atherosclerotic development, thus resulting in a StemBell-treated group containing five mice and a vehicle-treated group containing five mice. Blood samples were obtained within 3 days before and 3 days after treatment via the tail vein, and immediately prior to termination via cardiac punction, to determine blood glucose levels and circulating monocyte subsets. After termination, the heart and the connected thoracic aorta were excised for (immuno)histochemical analysis.

This study received approval on 19 December 2018 by the Central Committee for Animal Experiments of the Netherlands (the Centrale Commissie Dierproeven; AVD1140020186604) and was conducted in accordance with the institutional guidelines of the Animal Ethics Committee of the VU University, Amsterdam.

### 2.4. Tissue Processing

Tissue processing and experimental analyses were performed as described previously [20]. The dissected tissue was fixated in formalin and embedded in paraffin. Subsequently, the heart was separated into two parts: the upper part containing the aortic root and the remaining cardiac tissue. To accurately identify the location of the aortic root—defined as the initial segment of the aorta where the aortic valves reside—the upper heart section was serially cut into 4 μm-thick sections. Every 20th section underwent a haematoxylin-eosin (HE) staining. A total of 78 sections were then selected to ensure the entire aortic root (60 sections = 240 μm) was included. To prepare for analysis, every 13th section was systematically mounted onto a glass slide (i.e., slide 1 containing Section 1, 13, 26, etc.), generating 13 glass slides with 6 sections each. These slides provided a comprehensive cross-sectional view of the entire aortic root and were subsequently used for (immuno)histochemical analysis of plaque size, plaque stability, and intra-plaque inflammation.

### 2.5. (Immuno)histochemistry

Immunohistochemical staining was performed to analyse activated endothelium (ICAM-1), anti-inflammatory M2 macrophages (CD163), leukocytes (CD45), neutrophilic granulocytes (Ly6G), and pan-macrophages (Mac3). Table 2 depicts the specific procedures for each staining. First, the slides were deparaffinised in xylene for 10 min, rehydrated in graded ethanol (100–96–70%) for 10 min, and subsequently incubated with 0.3% H_2_O_2_ in methanol to block endogenous peroxidases. For the ICAM-1 staining, the slides were then incubated with graded ethanol (96–70–50%) for 3 min and rinsed with demi-water. Heat-inactivating antigen retrieval was performed by boiling tissue in citrate buffer (pH 6.0), or by incubating with 0.25% pepsin solution in 0.1 mmol/L HCl at 37 °C for 10 min. Blocking was achieved via pre-incubation with 1:10 normal mouse serum (NMS) or 1:50 normal rabbit serum (NRS) for 10 min, followed by incubation with the primary antibody for 1 h, or at 4 °C overnight. Antibodies were diluted in Tris-HCL-buffered saline (TBS) containing 1% BSA and 0.1% Tween (TBT) or in normal antibody diluent (NAD), and the incubations were performed at room temperature unless otherwise specified. For the CD163 staining, the slides were incubated with an antibody complex constructed by incubating mouse-anti-mouse CD163 antibody (Dako), rabbit-anti-mouse IgG antibody (Rockland), and NAD for 20 min, followed by addition of NMS for 10 min. After incubation with the corresponding secondary antibody for 30 min, the slides were incubated with a tertiary antibody where necessary for 30 min (ICAM-1 staining) or 1 h (CD45 and Ly6G staining). Subsequently the slides were visualised with 3,3′ diaminobenzidine (DAB; Dako) and counterstained with haematoxylin, dehydrated in graded ethanol (96–100%) and covered.

Plaque size and stability, as well as presence of pulmonary thrombi, were assessed on Haematoxylin-Eosin (HE)- and Elastica von Giesson (EvG)-stained slides [18], performed according to standard protocol. Analysis of mast cells was performed with a Toluidine Blue staining according to standard protocol. In short, tissue was first deparaffinised in xylene for 10 min and rehydrated in graded ethanol (100–96–70%) for 3 min. following a rinse with distilled water, tissue was incubated with Toluidine Blue (1:100, provided by Molecular Diagnostics Department, AUMC, Location AMC). Then, tissue was incubated with 70% ethanol for 15 s, dehydrated in graded ethanol (70–96–100%), and cleared in xylene before covering.

### 2.6. (Immuno)histochemical Scoring

To enable consistent analysis across experimental groups, measurements of plaque size and intra-plaque levels of inflammation, ceroid and fibrosis were conducted in the atherosclerotic plaques in the aortic root at the level of the aortic valves. The slides were digitised with a Phillips slide scanner (Amsterdam, The Netherlands). Plaque area (mm^2^) was quantified with Philips IntelliSite Pathology Solution v3.2 software (Amsterdam, The Netherlands) by measuring the surface area from the lumen to the media within each of the valve cusps. Plaques were included from the moment the valves opened until the valve was no longer visible. The mean plaque area per mouse was determined by averaging the measurements from the valve cusps. Intra-plaque ceroid and fibrosis content was assessed by encircling the atherosclerotic plaques in QuPath v.0.4.3 software on EvG-stained slides and transfering them to ImageJ v1.51g software, where intra-plaque fibrosis was selected with a colour threshold and intra-plaque ceroid area was encircled manually. Both areas were then measured and represented as percentage of the total plaque area. Endothelial ICAM-1 expression in aortic plaques was assessed as a percentage of the total aortic root endothelium. The intra-plaque inflammatory cell content was analysed digitally: QuPath v.0.1.2 software was used to select atherosclerotic plaques, then ImageJ v1.51g software was used to quantify total plaque area and positively stained areas. The area positive for an inflammatory cell marker was reported as the percentage of the total atherosclerotic plaque. Mast cells (Toluidine Blue-stained) were manually counted and reported as the number of cells/mm^2^ within the total atherosclerotic plaque.

### 2.7. PBMC Isolation

To determine the effect of StemBell therapy on the distribution of peripheral circulating monocyte subsets, blood samples were collected at three time points: within 3 days prior to treatment, within 3 days following treatment, and at the study endpoint 3 weeks following treatment. Pre- and post-treatment samples were collected by tail vein incision into LH-microvettes (Sarstedt, 16.443, Nümbrecht, Germany), whereas endpoint samples were collected by cardiac punction into 1.5 mL eppendorfs containing ethylenediaminetetraacetic acid (EDTA; Ambion, AM9260G, Hamburg, Germany) to prevent coagulation. To obtain the peripheral blood mononuclear cell (PBMC) fraction, all samples were processed the same day as follows. The pre- and post-treatment blood samples were diluted with 50 µL PBS due to the limited volume, whereas endpoint samples were processed without dilution. Samples were transferred onto Lymphoprep (Stemcell Technologies, 7801, Vancouver, BC, Canada) and centrifuged without brake at 1000× *g* at 4 °C for 20 min, after which the PBMC-containing layer could be aspirated. Diluted with PBS, the PBMC’s were centrifuged at 250× *g* at 4 °C for 10 min. The PBMC pellet was resuspended in 0.5–1 ml FBS supplemented with 10% DMSO, immediately frozen at −80 °C, and transferred to liquid nitrogen storage the following day.

### 2.8. Flow Cytometry

The blood samples collected at three time points—within 3 days before treatment, within 3 days after treatment, and 3 weeks post-treatment—were analysed with flow cytometry to assess the circulating monocyte subset distribution. Monocytes were first identified by CD11b positivity and subsequently classified into two main subsets based on Ly6C expression: Ly6C^hi^ (classical monocytes) and Ly6C^low^ (non-classical monocytes). Isolated PBMCs were thawed and resuspended in ice-cold fluorescence-activated cell sorting (FACS) buffer, consisting of PBS supplemented with 0.5% BSA, 0.02% NaN3, and 2 mmol/L EDTA. After the PBMCs were centrifuged at 600× *g* at 4 °C for 8 min, the PBMC pellet was resuspended in 250 µL FACS buffer and stained with 2.5 µL FITC-conjugated anti-CD11b antibody (eBioscience, Paris, France) and 2.5 µL APC-conjugated anti-Ly6C antibody (eBioscience, Paris, France). A 3 min incubation on ice was followed by addition of 1 mL FACS buffer and another centrifugation at 600× *g* at 4 °C for 8 min. Finally, PBMCs were resuspended in 200 µL FACS buffer for flow cytrometric analysis (BD FACSCanto™ II with FloJo v10.7.1 analysis software, San Jose, CA, USA).

### 2.9. Statistical Analysis

Data analysis was performed using Prism v.4.0 (Graphpad Software, La Jolla, CA, USA). Data distribution was assessed for normality with a Shapiro–Wilk normality test. Experimental group comparisons were assessed with an unpaired *t*-test (normally distributed data) or a Mann–Whitney test (non-normally distributed data). Within-group comparisons were conducted with a paired *t*-test (normally distributed data) or a Wilcoxon matched-pairs signed rank test (non-normally distributed data). Data values are displayed as mean ± standard deviation (SD), and *p* < 0.05 was considered statistically significant for all analyses.

## 3. Results

### 3.1. Body Weight and Blood Glucose Measurements

Body weight remained stable throughout the experiment in both the vehicle-treated and StemBell-treated mice, with no significant differences between, or within, treatment groups (Table 2).

Blood glucose measurements on day 10 post-STZ confirmed successful DM induction and insulin deficiency in both treatment groups: 20.8 ± 4.3 mmol/L in the vehicle-treated control mice and 18.3 ± 6.0 mmol/L in the StemBell-treated mice. In addition, both groups had elevated blood glucose measurements at pre- and post-treatment time points 8–9 weeks post-STZ compared to day 10 post-STZ, although only reaching significance within the StemBell-treated mice (*p* = 0.03). The mice did not receive any insulin treatment in the meantime. Between the treatment groups there were no differences in blood glucose levels found at the different time points.

### 3.2. StemBell Therapy Complications

**Figure 3:**
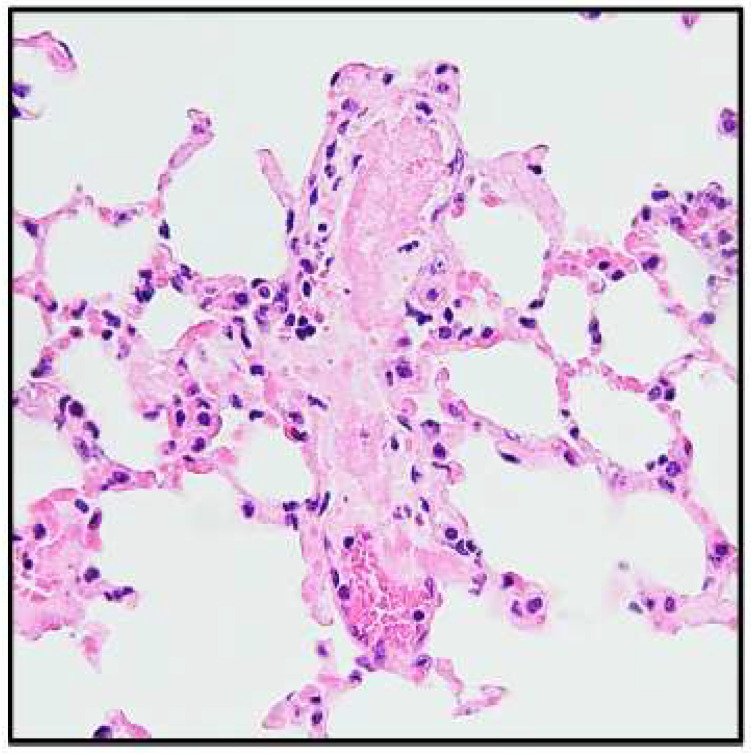
Example of pulmonary thrombus in lung tissue of a mouse deceased after StemBell treatment. The three mice that showed respiratory distress and died within 3–5 min after injection with 5 × 10^5^ StemBells were autopsied directly post-treatment. Presence of pulmonary thrombi was assessed with a Haematoxylin and Eosin (H&E) staining (original magnification ×200).

StemBell therapy was applied in diabetic mice 8 weeks after the first STZ injection to assess the effect on early atherosclerotic plaques (Figure 1). Initially, we injected 5 × 10^5^ StemBells per mouse, the amount that was used successfully in the previous study without drop-out or adverse effects [18]. However, immediately after injection with 5 × 10^5^ StemBells, the first three mice showed visible breathing distress and respiratory collapse, resulting in death within 3–5 min post-injection (3/3†). Presence of pulmonary thrombi was indicated in an H&E staining of the lung tissue of these deceased mice (Figure 3). In contrast, the vehicle-treated mice did not show these effects. Using immunohistochemistry, we also verified whether the pulmonary vasculature of DM mice had higher expression levels of ICAM-1—the target of the StemBells—than non-DM mice, which theoretically could result in more intraluminal StemBell aggregation in the lungs. To this end, we compared ICAM-1 expression in the lung tissue of 2 DM mice that died of respiratory collapse to that of 3 non-DM mice that we used in a previous StemBell study [18]. High expression levels of ICAM-1 were found in the lungs of both non-DM and DM mice, including in the vasculature, without obvious differences between them (Supplemental Figure S1).

Due to these unforeseen detrimental effects, the experiment was halted and, after careful consultation with the animal ethics committee, restarted a week later using 2.5 × 10^5^ StemBells per injection. As the first injection with StemBells again resulted in complications (1/1†), the concentration was immediately reduced to 1.25 × 10^5^ StemBells per mouse. Except for one mouse, the remaining StemBell-treated mice survived without any adverse effects observed (1/7†).

### 3.3. Atherosclerotic Plaque Features

**Figure 4:**
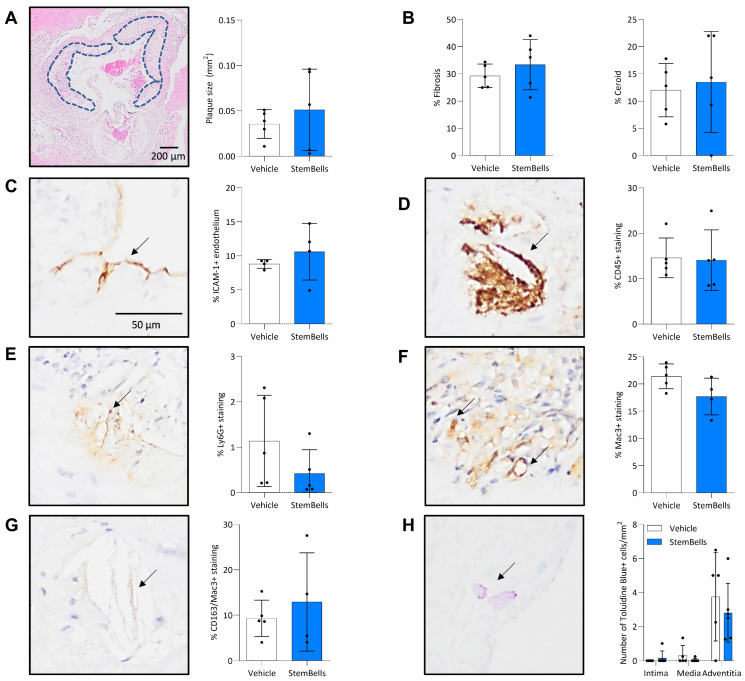
Atherosclerotic plaque characteristics in diabetic ApoE^−/−^ mice treated with vehicle or StemBells. Atherosclerotic plaque size (mm^2^) was assessed with an EvG staining of the aortic root ((**A**); original magnification ×40). Plaques were included from the moment the aortic valves opened (encircled in blue) to the moment the aortic valves were no longer visible. Plaque stability was assessed via quantification of intra-plaque fibrosis and ceroid (**B**). Intra-plaque inflammation was analysed through immunohistochemical staining, as indicated with arrows in a vehicle-treated mouse ((**C**–**H**); original magnification ×200). The percentage of ICAM-1+ endothelium (**C**), intra-plaque leukocytes (CD45; (**D**)), neutrophilic granulocytes (Ly6G; (**E**)), intra-plaque pan-macrophages (Mac3; (**F**)), intra-plaque M2 macrophages (CD163) as percentage of Mac3 staining (**G**), and the number of Toluidine Blue+ mast cells in the intima, media, and adventitia of the aortic root (**H**), were quantified in all mice. An unpaired *t*-test was used for analysis, data is presented as mean ± SD with statistical significance defined as *p*-value < 0.05.

All mice, except one, developed atherosclerotic plaques in the aortic root (Figure 4A). There was no significant difference in mean plaque size between the vehicle-treated (0.04 ± 0.02 mm^2^) and the StemBell-treated mice (0.05 ± 0.04 mm^2^, *p* = 0.5) (Figure 4A). Similarly, there was no significant difference in the mean percentages of intra-plaque fibrosis (*p* = 0.4) or ceroid (*p* = 0.8) (Figure 4B), mean percentage ICAM-1+ endothelium (*p* = 0.3) (Figure 4C), or mean percentage intra-plaque CD45+ staining (leukocytes; *p* = 0.9) (Figure 4D). In contrast, the mean percentage of Ly6G+ staining (neutrophilic granulocytes; 0.4 ± 0.5%, *p* = 0.2) (Figure 4E) and Mac3+ staining (pan-macrophages; 17.7 ± 3.4%, *p* = 0.09) (Figure 4F) was decreased compared to vehicle-treated mice (1.1 ± 1.0%; 21.4 ± 2.3%, respectively), albeit without reaching statistical significance. Meanwhile, StemBell-treated mice had a non-significant higher percentage of the CD163+ anti-inflammatory M2 macrophage subpopulation (of the total Mac3+ macrophages; 12.9 ± 10.8%, *p* = 0.5) (Figure 4G) than vehicle-treated mice. The number of mast cells in the media and intima of the aortic root did not differ between experimental groups, but the number of adventitial mast cells was decreased in StemBell-treated mice (2.9 ± 1.9, *p* = 0.6) compared to vehicle-treated mice, although non-significantly (Figure 4H).

### 3.4. Circulating Monocyte Subsets

**Figure 5:**
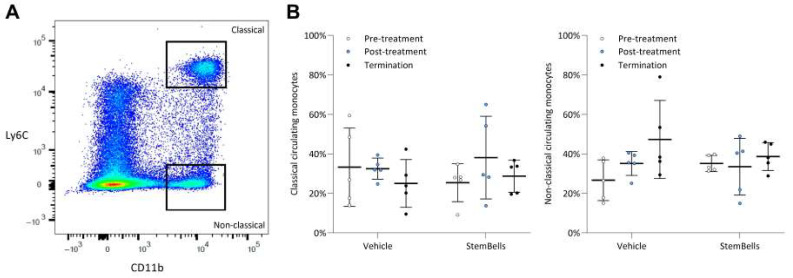
Peripheral circulating monocyte subsets in diabetic ApoE^−/−^ mice treated with vehicle or StemBells. Flow cytometric analysis (**A**) was performed on PBMCs collected within 3 days pre- and post-treatment, and at study endpoint (**B**), to evaluate peripheral circulating monocyte subsets. Ly6C^hi^ (classical) and Ly6C^low^ (non-classical) monocytes are presented as percentages of Ly6C+ monocytes. Statistical comparisons between treatment groups were assessed with an unpaired *t*-test, and within treatment groups with a paired *t*-test (termination vs. pre-treatment measurements and vs. post-treatment measurements). Data is presented as mean ± SD with statistical significance defined as *p*-value < 0.05.

Figure 5A shows the distribution of classical and non-classical monocytes in blood samples collected within 3 days prior and 3 days post-treatment and at termination (21 days post-treatment).

Prior to treatment, the percentage of pro-inflammatory classical monocytes was similar in the vehicle-treated (33.2 ± 19.9) and StemBell-treated group (25.1 ± 8.6) (Figure 5B). In the vehicle-treated mice, the percentage of classical monocytes did not differ 3 days post-treatment, but seems slightly decreased at termination 21 days post-treatment (25.0 ± 12.1, *p* = 0.6), although this was not significant. In contrast, in the StemBell-treated mice the percentage of classical monocytes was increased three days post-treatment (38.1 ± 20.9, *p* = 0.3), although non-significantly, whilst 21 days post-treatment the percentage was again similar to the percentage at pre-treatment (28.7 ± 8.1).

The percentage of anti-inflammatory non-classical monocytes was also similar prior to treatment in the vehicle- (33.2 ± 19.9) and StemBell-treated mice (25.4 ± 9.6) (Figure 5B). In the vehicle-treated mice, the percentage of non-classical monocytes seemed slightly increased 3 days post-treatment (35.1 ± 6.1, *p* = 0.1) and 21 days post-treatment (47.3 ± 19.8, *p* = 0.2), although without reaching statistical significance. In contrast, in the StemBell-treated mice the percentage of non-classical monocytes remained similar both 3 days post-treatment (33.46 ± 14.4) and 21 days post-treatment (38.6 ± 7.1).

Taken together, our findings show that there were no significant differences within the experimental groups, nor between the experimental groups.

## 4. Discussion

The aim of this project was to validate targeted StemBell technology in a streptozotocin-induced DM mouse model as a potential novel therapeutic tool to treat macrovascular inflammation in patients with DM. Unfortunately, we found that StemBell injection with 5 × 10^5^ and 2.5 × 10^5^ StemBells led to respiratory collapse and death within 3–5 min post-injection in mice with diabetes mellitus and early atherosclerosis. With a reduced dose of 1.25 × 10^5^ StemBells, we observed no significant alterations in intra-plaque fibrosis, ceroid, and inflammation (i.e., macrophages, neutrophils, and mast cells) in the StemBell-treated mice compared to the vehicle-treated mice.

Targeted StemBell therapy previously showed beneficial effects on plaque stability through decreased inflammation markers and increased fibrotic cap thickness, without affecting plaque size, in mice post-MI [1]. In that study, there was no mortality after injection with 5 × 10^5^ StemBells in mice with established atherosclerotic plaques; therefore, no adverse effects were expected for this project. However, we found that injection with 5 × 10^5^ StemBells led to respiratory collapse and death in diabetic mice with established atherosclerotic plaques representative of early atherosclerosis. Consequently, the number of StemBells had to be drastically reduced to 1.25 × 10^5^ StemBells to avoid respiratory complications. Autopsy on the deceased mice indicated the presence of pulmonary thrombi, which may have blocked the vessels thus resulting in the observed respiratory collapse. This notion is corroborated by the findings of Frodermann et al. [13], who found fluorescently labelled MSCs to be mainly accumulated in the lungs 15 min after intravenous injection in diabetic LDL^−/−^ mice. As the StemBells were targeted to ICAM-1, we hypothesised that higher expression levels of ICAM-1 in the pulmonary vasculature of DM mice could result in more intraluminal StemBell aggregation in the lungs. We found already high expression levels of ICAM-1 in the lung vasculature of non-DM mice, as has been shown before [24], which was similar to that in the lungs of the DM mice. Indeed, it has been shown recently that presence of ICAM-1 on the luminal surface of endothelial cells in the lungs of C57Bl/6J mice was not affected by DM [25]. Therefore, we cannot conclude that the observed respiratory collapse after StemBell administration in DM mice is related to a difference in ICAM-1 expression in the lungs. Another cause could be the adjusted vascular structure in DM, for instance increased vascular stiffness, which may result in the inability to pass the StemBells. Particularly, the complex seems to be the issue, as the application of stem cells [20] and microbubbles (not yet published) separately did not result in mortality. We agree that it is essential to further explore the options, perhaps even use a larger animal model to see if a larger vessel size can mitigate the issues of aggregation and blockage [26].

The lower StemBell dose may have reduced the therapy’s efficacy. Albeit, in clinical trials of intravenous stem cell therapy around 1–4 million stem cells/kg bodyweight are applied [27,28,29,30,31], which translates to approximately 25,000–100,000 stem cells in mice. The dose of 1.25 × 10^5^ StemBells thus is proportionate to clinically applied doses. The lack of therapeutic effect of StemBell therapy we found in this study is in line with a previous study in atherosclerotic hyperglycaemic mice, in which a clinically relevant dose of intravenous ASC did not induce significant effects on plaque characteristics [20]. Indeed, the differences in stem cell doses between human trials and murine studies, in which converted stem cell doses multiple times higher than in patients are commonly given, may explain the differences in stem cell therapy effects between clinical and murine studies.

In addition to the low cell dose, the efficacy of the StemBell therapy may have also been affected by the diabetic environment of our mouse model. Among its various deleterious effects, hyperglycaemia can alter the paracrine signalling mechanisms of ASC/MSC. Previous studies (both murine and clinical) found that type 2 DM-isolated ASCs exhibited a more inflammatory profile [32,33,34,35]. Additionally, hyperglycaemia may induce MSC senescence and diminish crucial functions of MSCs, such as proliferation and differentiation, angiogenesis, and immunomodulatory capability, which remarkably restrict their therapeutic efficiency [36]. Our approach differed by isolating ASCs from non-DM mice for therapeutic application in diabetic mice. However, in vitro studies show that exposure of non-DM ASCs to conditioned medium of type 2 DM ASCs [37] or to hyperglycaemic conditions [38,39] can also result in an inflammatory profile as observed in DM ASCs. DM-induced inhibition of therapeutic ASC activity may thus in part be a cause of the limited effect of StemBell therapy on atherosclerotic plaque characteristics in diabetic mice as observed in this study. However, the effect of a diabetic environment on ASC function may be mitigated by increasing treatment frequency. For instance, Yu et al. showed that multiple ASC infusions improved DM-induced complications, including cardiac function, in a DM rat model via alleviating inflammation and promoting tissue repair [40]. Furthermore, Saleh et al. found that ASC therapy with two timepoints alleviated obesity-related complications in obese mice, which actually developed DM during the experiment [41]. While there are no human studies with ASC therapy in DM patients yet, these pre-clinical findings in combination with currently running clinical trials on ASC therapy in non-DM patients are encouraging [42].

## 5. Conclusions

In this study, we aimed to investigate the effect of StemBell therapy on atherosclerotic plaque characteristics in a streptozotocin-induced DM mouse model. Unexpected mortality of mice led to a 4-fold lower dose of StemBell therapy, resulting in less mice to study. Therefore, we cannot draw definitive conclusions from our findings as to whether StemBell therapy has the potential to improve atherosclerotic plaque stability in a diabetic environment. To assess the possibilities and limitations of StemBell therapy in DM models, further research is warranted in order to improve StemBell therapy for clinical use.

## Figures and Tables

**Table 1:** Changes in bodyweight and blood glucose measurements of included mice. Bodyweight (grams) was measured 2–3 a week for the entire study duration: initial streptozotocin injection (start) to study endpoint (end). Blood glucose levels (mM) were measured at four timepoints: initial streptozotocin injection (start), within 3 days pre-treatment, within 3 days post-treatment, and study endpoint (end). Comparisons were made between treatment groups on the same day (not significant), and within treatment groups over time (* displays significant difference from the start value). Data is displayed as mean ± SD. *p* < 0.05 *.

Treatment	Body Weight (g)		Blood Glucose (mmol/L)		
	Start	End	Verification	Pre-treatment	Post-treatment
Vehicle	24.8 ± 1.6	22.3 ± 3.4	20.8 ± 4.3	27.8 ± 0.0	24.2 ± 8.1 *
StemBells	24.0 ± 1.4	23.7 ± 3.5	18.3 ± 6.0	24.5 ± 6.8	27.8 ± 0.0 *

**Table 2:** Specification of immunohistochemical staining procedures. Immunohistochemical staining was performed to analyse activated endothelium (ICAM-1), anti-inflammatory M2 macrophages (CD163), leukocytes (CD45), neutrophilic granulocytes (Ly6G), and pan-macrophages (Mac3). Abcam (Amsterdam, The Netherlands); BD Biosciences (San Jose, CA, USA); Dako (Glostrup, Denmark); Immunologic (Arnhem, The Netherlands); Jackson ImmunoResearch (Ely, UK); Vector Laboratories (Neu-Isenburg, Germany).

Target	Marker	Antigen Retrieval	Blocking Serum	Antibody Solvent	Primary Antibody Reference	Secondary Antibody Reference	Tertiary Antibody Reference	Visualisation (min)
Activated endothelium	ICAM-1	Citrate	-	TBT	1:500 Abcam, 119871	1:400 Dako, E0468	1:100 Vector Laboratories, PK6000	1–3
Anti-inflammatory M2 macrophages	CD163	Citrate	NMS	NAD	1:200 Dako, M0794 Rockland, 810-4102	1:50 Immunologic, DPVR-120HRP	-	7–10
Leukocytes	CD45	Citrate	NRS	NAD	1:50 BD Biosciences, 553076	1:100 Jackson ImmunoResearch, 212-065-04	1:100 Dako, P0397	7–10
Neutrophilic granulocytes	Ly6G	Pepsin	NRS	NAD	1:200 BD Biosciences, 551459	1:100 Jackson ImmunoResearch, 212-065-04	1:100 Dako, P0397	7–10
Pan-macrophages	Mac3	Citrate	NRS	NAD	1:30 o/n BD Biosciences, 553322	1:50 Dako, P0162	-	7–10

## Data Availability

All data relevant to the study are included in the article.

## Supplementary Materials

The following supporting information can be downloaded at: https://www.mdpi.com/article/10.3390/biology14091130/s1, Figure S1: Example of ICAM-1 expression in lung tissue of non-DM and DM mice after StemBell treatment. We compared ICAM-1 expression in the lung tissue of 2 DM mice that died of respiratory collapse to that of 3 non-DM mice that we used in a previous StemBell study [18]. ICAM-1 expression was assessed with immunohistochemical staining (original magnification ×40).
