# Supplementary material for: StemBell Therapy Does Not Significantly Affect Atherosclerotic Plaque Characteristics in a Streptozotocin-Induced Diabetes Mellitus Mouse Model
Source: Biology (Basel). 2025 Aug 26;14(9):1130. doi: 10.3390/biology14091130 (PMC12467836; doi:10.3390/biology14091130)
Supplement: Supplementary file 1 [file biology-14-01130-s001.zip › biology-3726292-supplementary.pdf]

biology

MDPI

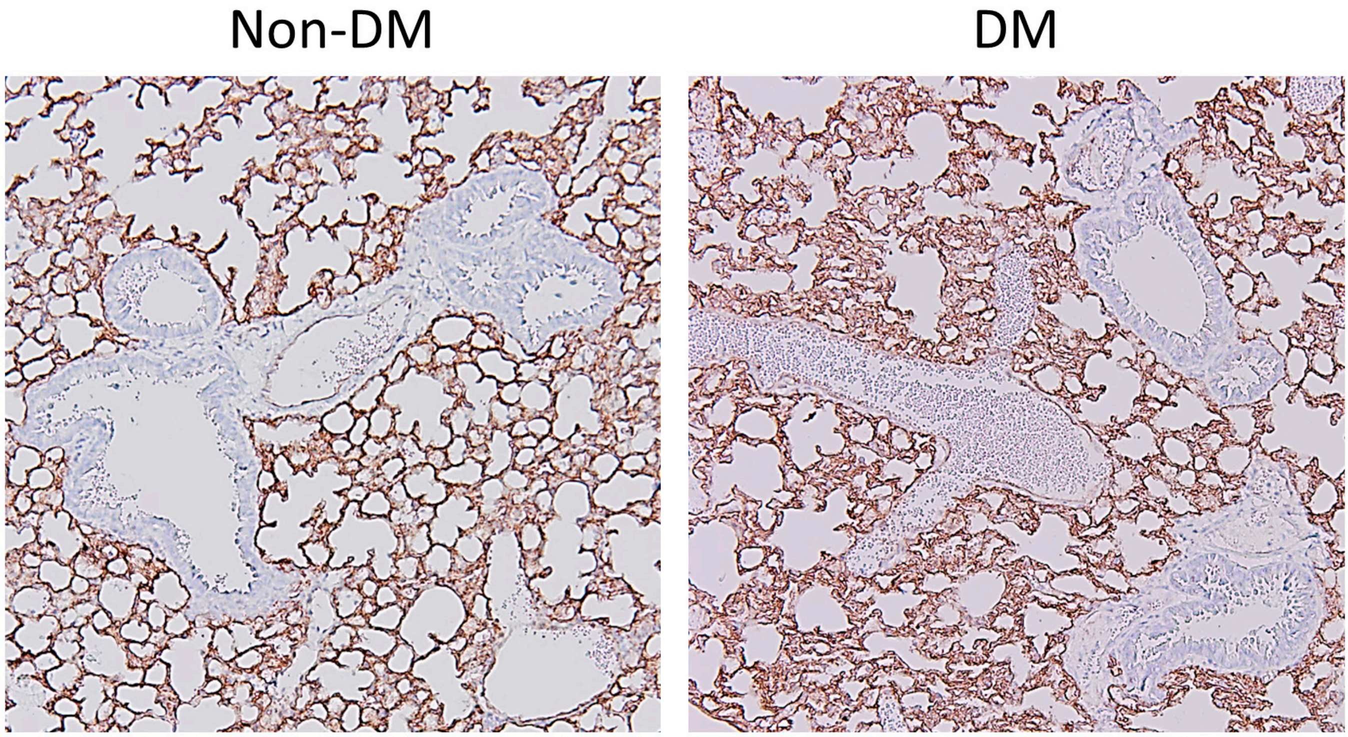

Supplemental Figure S1. Example of ICAM-1 expression in lung tissue of non-DM and DM mice after StemBell treatment. We compared ICAM-1 expression in the lung tissue of 2 DM mice that died of respiratory collapse to that of 3 non-DM mice that we used in a previous StemBell study [18]. ICAM-1 expression was assessed with immunohistochemical staining (original magnification × 40).
